# Clinicians’ perspectives on machine learning for obstructive sleep apnoea detection: a human factors study

**DOI:** 10.3389/fdgth.2026.1878569

**Published:** 2026-08-05

**Authors:** Abdelrahman Otify, Ian Nabney

**Affiliations:** 1 Centre for Doctoral Training in Digital Health and Care, University of Bristol, Bristol, United Kingdom; 2 School of Engineering Mathematics and Technology, University of Bristol, Bristol, United Kingdom

**Keywords:** clinicians, human factors, machine learning, obstructive sleep apnoea, qualitative study

## Abstract

Obstructive sleep apnoea is a common sleep disorder affecting 5%–15% of the population, with many going undiagnosed due to accessibility issues and long waiting lists. This is due to the bottleneck method of diagnosis, which is polysomnography. The implementation of machine learning, particularly when applied to a reduced set of physiological signals, has the potential to enhance accessibility and to mitigate the demands associated with conventional polysomnography, a procedure that is inherently time-consuming and labour-intensive. In this preliminary study, 10 clinicians were interviewed to gather their perspectives on the use of machine learning in the domain. Using thematic analysis, key themes were identified, highlighting important considerations on the deployment of machine learning. Findings suggest the need for guidelines and approvals to regulate the use of machine learning, the importance of including medical experts in the process to ensure the best healthcare service is provided and that clinicians lead and not machine learning. The aim is to further the understanding of the complexities of machine learning and understand how to better tailor machine learning to allow for the adoption of machine learning and increase the trust in machine learning by clinicians.

## 1 Introduction

Obstructive sleep apnoea is a sleep-related breathing disorder that occurs due to repeated obstruction of the upper airway, the throat (1). The gold-standard method to diagnose obstructive sleep apnoea is polysomnography (PSG) (2). It monitors patients’ physiological signals, and it requires supervision by a trained technician during the patient’s sleep. It also requires manual scoring of the polysomnogram. The scoring of PSG usually takes 1–2 h (3). This makes PSG a labour-intensive and expensive procedure. The use of machine learning would speed up the process of obstructive sleep apnoea (OSA) detection because machine learning is good at detecting patterns, and it would also be good at analysing the complex signals which are produced by polysomnography.

In theory, machine learning algorithms have shown that they can have high performance in the detection of OSA (4). For example in the work by Padovano et al., where accuracy has reached 85.46%. However, the performance of machine learning algorithms must be shown in the real world in clinical settings. It is also important to understand the perspectives of the users on machine learning algorithms and whether they would like to adopt machine learning systems in a clinical setting. It is also important to know whether it would be possible to involve machine learning and artificial intelligence (AI) usefully in clinicians’ diagnosis workflow and whether they would trust it. The use of machine learning or AI can often be seen as total automation and completely eliminating human input. However, it is important to understand how and whether clinicians would be happy to work alongside machine learning systems, so they can be aided using this tool. It is not just essential for machine learning to have a high predictive performance, but it is also important that machine learning outputs are useful in the clinical setting. Light needs to be shed upon the fact that machine learning has been used as a tool that signals are fed into, and it outputs an outcome. But, in a clinical setting, the story is different, a clinician gathers patient history, evaluates symptoms and orders specific diagnostic tests to reach a definitive diagnosis (5). The approach is more holistic. So, it is very important that the difference in diagnosis is considered and that clinicians are asked about their concerns regarding this approach.

Therefore, the main objective of this study is to qualitatively explore clinicians’ perspectives on the use of machine learning for OSA detection. Specifically, this work aims to identify the requirements to allow clinicians to trust machine learning detection algorithms, gauge clinicians’ willingness to adopt machine learning algorithms and understand their concerns regarding diagnostic methods that rely on reduced physiological signals. Our results indicate that while clinicians recognise the potential of machine learning, they view patient history-taking as an irreplaceable gateway to diagnosis, requiring AI to act as a supportive tool requiring human oversight rather than a fully automated replacement. Furthermore, clinicians expressed mixed opinions on using machine learning models that analyse fewer signals; while potentially more efficient, they warned against this approach for patients with complex medical history and comorbidities due to overlapping symptoms and signal artefacts. Ultimately, successful clinical adoption of machine learning in OSA detection requires a holistic picture that utilizes familiar metrics like the Apnoea-Hypopnoea Index (AHI), alongside clear regulatory guidelines to ensure clinical safety and trust.

## 2 Background

To understand the context of this study, it is important to first establish the theoretical framework for applying machine learning to OSA detection. Typically, machine learning methods ranging from traditional classifiers like Support Vector Machines, Random Forests and Deep Learning architectures are trained on physiological signals extracted from PSG, such as ECG, oxygen saturation, and airflow (6). These algorithms are designed to detect patterns, classify respiratory events, or predict the AHI (6). Crucially, these machine learning methods are designed to function as clinical decision support systems. They are used to generate a preliminary diagnosis, flag high-risk patients, or reduce and remove the burden of manual scoring of PSG data, but they do not exclude the clinician’s involvement. The clinician remains essential for evaluating complex patient histories, reviewing the AI’s preliminary findings, and making the final definitive diagnosis.

Some of the ideas in the introduction have been explored in the literature, albeit in other fields of research and not in the area of obstructive sleep apnoea. Gu et al. in (7) have shared the lessons learnt from the designing of an AI tool, Impetus, to aid pathologists in the detection of tumours from histological slides. Only 8 pathologists have been studied in (7). The lessons learnt as reported from the study are that an AI system should allow the medical user to see the evidence that lead to a certain recommendation. This relates to the interpretability and explainability of AI. It is important that AI does not increase or add any administrative burdens and should help to minimise it. It should integrate seamlessly into clinical workflows. A balance needs to be struck between the user effort required to use the AI system and the clinical actionability of the resulting insights. The issue of time also came up in (8), participants mentioned that it would be quicker to diagnose a patient without the AI-powered clinical decision support system. Another study (9) in the area of pathology and the use of deep learning for predicting prostate cancer diagnosis, a study that involved 21 pathologists. The main information desired were the machine learning overall performance, including strengths and limitations. What information does the machine learning system has access to, and how it uses the information to predict. Also, clinicians wanted information on regulatory approval, peer-reviewed sources validating the machine learning tool, legal liability and impact on clinical workflows. In another study, machine learning explanations that supported transparency at decision points were reported as unhelpful; however, it was only needed by the clinicians if the outputs of the machine learning were seen as surprising (5). It is important to note that clinicians abandoned the machine learning outcome if the output did not align with clinician knowledge and guidelines. Also, in (8), clinicians wanted to understand how the AI system produces recommendations for each patient, which would allow them to trust the system. Also, clinicians were interested in the scientific and clinical basis upon which the system diagnoses patients and generates medical advice. They wanted this so they could evaluate whether the system’s recommendations are appropriate.

The idea of AI integration into clinical workflows have been discussed as well by Beede et al. in (10). This was in a system that uses deep learning to assess diabetic retinopathy, which is a condition caused by high blood sugar that damages the blood vessels in the retina. The idea of using the machine learning system is to assist nurses and to eliminate the need for nurses to send images to ophthalmologists for review. This would essentially remove the bottleneck that would provide patients with immediate results, and it would enable nurses to make a referral recommendation in the moment. This subsequently allows for faster facilitation of treatment, would allow more cases to be screened and would allow nurses to spend more time with patients on diabetes management and education, which can improve patient outcomes. In that study, the nurses wanted to see the pictures taken for each patient that was used in the machine learning, and the prediction from the machine learning was not enough. This helped in things like ensuring it was the correct picture of the retina for the patient, and it would also help the nurses with information that can convince patients to seek treatment. Also, nurses wanted to apply their learning and develop their experience by viewing the pictures. Also, pictures of the retina and the prediction from the machine learning system would help empower the nurses, as their diagnosis can sometimes be dismissed by more senior staff. Nurses were worried that using the machine learning system added to their workload, given the volume of patients. They were also worried about extra test and treatment costs, travel burden and missing work, if the result is a false positive.

Research shows that clinicians have very different views on using AI. Some want to understand exactly how the machine learning systems make its decisions. Others aren’t as worried about the technicalities; they just want to know if the system is legal, safe, and officially approved. Finally, there is a group that just wants to know if the AI works. For them, the most important thing is that the predictions are accurate and the machine learning system’s performance is high. Drawing from the existing literature and the theoretical understanding that machine learning serves as a preliminary tool rather than a replacement for clinicians, the semi-structured interview guide was developed to evaluate clinician perspectives on the integration of machine learning for OSA detection.

## 3 Methods

To understand the perspectives of clinicians on the use of machine learning for the detection of OSA, interviews were conducted. The aim of the qualitative study is to capture the opinions and thoughts of clinicians who work and diagnose patients with OSA. The study was approved by the Faculty of Engineering Research Ethics Committee at the University of Bristol.

### 3.1 Data collection

Participants who were clinicians who worked with patients who were suspected of OSA were included in the study. A total of 10 participants were interviewed. To ensure transparency and allow for the transferability of the findings, detailed participant characteristics are summarised in Table 1.

**Table 1:** Participant characteristics and demographics.

ID	Specialty/role	Healthcare setting
P1	Respiratory & Sleep Medicine/Consultant & University Professor	Hospital & Clinic
P2	Respiratory/Doctor	Hospital & Clinic
P3	Sleep Medicine/Consultant & University Professor	Hospital & Clinic
P4	Respiratory/Intensive Care Unit Doctor	Hospital
P5	Ear, Nose and Throat/Doctor & Academic Research	Hospital & Clinic
P6	Neurologist/Consultant	Hospital
P7	Respiratory/Doctor & Academic Research	Hospital
P8	Sleep Medicine/Consultant	Hospital
P9	Neurology/Doctor	Hospital
P10	Neurology & Sleep Medicine/University Professor & Academic Research	Hospital

The interviews included main questions with additional sub-questions to help with probing and to gain more in-depth information. The main questions were:

- How do you diagnose obstructive sleep apnoea currently?
- Do you feel that all the signals from polysomnography are needed in obstructive sleep apnoea detection?
- What is your understanding/view on machine learning?
- What is your general feeling about using Artificial Intelligence/machine learning to detect obstructive sleep apnoea?
- What is your perspective into how machine learning algorithms should output the results?
- Do you use machine learning at all in your job?
- Do you think machine learning can make your job more effective or easier? If so, how?

Interviews were recorded on Microsoft Teams, and the interviewer would take notes to help with the analysis after the interview and to ask additional questions if necessary to gain more understanding of anything that was said by the participant. Interviews ranged from 15 to 25 min, and the interviews were transcribed verbatim.

### 3.2 Analysis

The entire data corpus was analysed using thematic analysis, following the widely established six-phase methodological framework outlined by Braun and Clarke (11). The analysis was driven by the data rather than pre-existing theories, with a specific focus on capturing clinicians’ perspectives regarding the use of machine learning for OSA detection and screening.

To ensure transparency and rigour, the coding process was conducted systematically. First, the transcripts were read iteratively for data familiarisation by the first author. The first author then conducted the initial coding by highlighting statements of interest and generating descriptive codes directly from the raw data. This coding process was managed using Microsoft Excel.

Following initial code generation, the codes were examined for patterns and collated into potential overarching themes. To enhance the reliability of the findings, the primary author cross-referenced the themes against the original transcripts to ensure they accurately reflected the clinicians’ perspectives. This iterative process of theme development and interpretation allowed the final themes to emerge organically from the data.

## 4 Findings

Using insights gained from the interviews, 3 main themes were generated: “History-Taking as the Gateway to Sleep-Disorder Diagnosis”, “Balancing Signal Depth with Patient Complexity”, and “We want the picture to be clear and not hazy”.

### 4.1 History-taking as the gateway to sleep-disorder diagnosis

It was evident across the board from the different participants that history taking and analysing the symptoms of the patient was a key step in the diagnosis process: “A patient will have some symptoms, but we cannot confirm until the patient has went through a sleep study”(P6). Due to the nature of OSA and the fact that it occurs during sleep, it was also mentioned by one of the participants that they would ask the sleep partner about the patient’s sleep: “The partner witnessed apnoea episodes and they described the event”(P1) and based on the sleep partner witnessing the episodes, the clinician could not rule out OSA. The patient was suspected of OSA, and they were referred to undertake a sleep study. Clinicians would also look at: “physical characteristics of the patient and the phenotype and then perform a PSG”(P5). All these are evidence that even if machine learning algorithms were to be deployed, clinicians or someone with medical expertise would be needed to direct and refer patients to sleep studies and further testing. However, it was clear from the participants that the final, definitive diagnosis would be made by the medical test, which in this case is the sleep study.

These perspectives likely stem from the foundational medical principle of treating the patient. Because OSA is deeply intertwined with qualitative lifestyle factors, anatomical features, and third-party observations, such as sleep partners. Clinicians recognise that the data required to contextualise sleep data cannot currently be captured by physiological sensors alone. Human-gathered context is essential, and without it, clinicians feel that machine learning outputs lack the clinical reality needed to make safe, individualised care decisions. In addition to all of the above, it is clear that all the decisions are initially made by the clinicians. This is similar to what appeared in (8), where clinicians wanted to rely on their knowledge and experience to diagnose the patients rather than being guided and directed by the artificial intelligence-powered clinical decision support systems. It was also shown in (12) that artificial intelligence can struggle with the complex medical history of patients. Therefore, the oversight of medical expertise is important whenever machine learning is deployed.

For the future use of machine learning in clinical practice, these findings suggest that AI systems should not be designed as standalone diagnostic systems that attempt to bypass initial clinical assessments. Instead, they must be strictly used as assistive tools. Algorithm developers and researchers must design systems that seamlessly integrate the quantitative outputs of machine learning alongside the qualitative patient history gathered by the clinician. Therefore, the oversight of medical expertise is not just a regulatory formality, but it is an important part of the diagnosis process whenever machine learning is deployed.

### 4.2 Balancing signal depth with patient complexity

Participants were asked about the application of machine learning on fewer signals than the number of signals in a polysomnography (PSG), or what is commonly known as a sleep study. For example, they would be asked about applying machine learning to ECG signals or oxygen saturation on their own without other signals. There was a general split in opinions, as some agreed “as long as the diagnostic performance is the same (as the gold-standard)”(P5). Others opposed the idea as they mentioned that usually “the artefacts are already too much with the full sleep study”(P1), which means that if the process relies on 1 signal, and the signal is not good enough due to artefacts, then the process would have to be repeated, and the detection would not be reliable. Therefore, repetition of the detection process would be needed. This would make the diagnosis lengthy and inefficient. This aligns with the work by Yang et al., where it was reported that decision support tools can be disruptive and time-consuming (13). This concern stems from the practical realities of sleep monitoring; sensors frequently detach or degrade due to patient movement, and a single-point failure in a setup which uses reduced signals can render the entire detection attempt useless. For future machine learning applications, this means algorithms must not only classify apnoeic events but also incorporate automated signal quality assessments to flag data with lots of artefacts immediately, thereby minimising the need for costly repeat tests.

It was also mentioned that a certain healthcare system uses these limited-channel testing devices as a preliminary indicator because a full polysomnography cannot be conducted due to “cost restraints and that pulse oximetry is cheaper”(P8). And, if the “results from the pulse oximetry is not clear, results do not correspond with clinical suspicion or are indicative of obstructive sleep apnoea, the patient is referred for a polysomnography”(P9). This shows that the use of limited-channel testing can be used for healthcare resource management and a primary cost-effective screening tool rather than a definitive clinical endpoint in the clinical diagnostic pathway. It is a way to reserve resource-intensive polysomnography for ambiguous, complex and highly suspicious cases. Consequently, future machine learning models utilising limited channels should be explicitly designed and regulated as triage tools rather than diagnostic replacements. Their primary clinical utility will be in rapidly clearing low-risk patients and prioritising high-risk patients for full PSG, directly addressing healthcare backlogs.

Regarding the patient history and co-morbidities, participants mentioned that reduced signals would be sufficient when the patient is “healthy with no co-morbidities”(P2). This is sensible since OSA can overlap with conditions like Cheyne-Stokes, for example. P2 mentioned an example: “If you use oxygen saturation, how will you differentiate between pulmonary embolism and OSA? Especially since pulmonary embolism comes and goes and is on and off”, suggesting the similarity between pulmonary embolism and OSA. There will be no way to differentiate between the two conditions using just one signal. This overlap is not limited to just respiratory conditions or sleep disorders; P2 asked: “If you use heart rate, how will you differentiate between OSA and anxiety?” This shows that conditions affecting mental health and wellbeing can affect breathing, but the cause has nothing to do with sleep disorders. Clinicians express these concerns because isolated physiological signals are inherently non-specific; a drop in oxygen or a spike in heart rate is a shared symptom across numerous cardiovascular, pulmonary, and psychiatric conditions. Moving forward, the deployment of reduced-signal machine learning systems must be strictly governed by clinical exclusion criteria, ensuring they are only utilised for patients with straightforward, uncomplicated medical histories to avoid dangerous misdiagnoses.

P1 and P8 were not satisfied with having a reduced number of signals for the detection of obstructive sleep apnoea, as “How can you ensure that the supposed episode has actually happened during sleep without EEG?”(P1) and P8 mentioned that “most people don’t know about Respiratory Effort-Related Arousals (RERAs) and they can only be detected with EEG and all the other channels on full polysomnography … so I always feel worried”(P8). These concerns highlight a critical limitation of reduced-channel devices that do not include EEG to accurately differentiate sleep from wakefulness or for the detection of the RERAs. Therefore, these systems might underestimate the disease severity or prevent the clinician from knowing the holistic picture of the patient’s disease profile. Ultimately, developers must ensure that future machine learning systems transparently communicate their clinical limitations, explicitly stating what the algorithm cannot detect, such as RERAs. This will prevent clinicians from being given a false sense of diagnostic certainty when using reduced-signal technologies.

### 4.3 “We want the picture to be clear and not hazy”

Participants were asked about the use of machine learning in general in the medical setting and in the field of OSA. One of the participants mentioned that they “want the picture to be clear and no hazy”(P2), and this summarised a lot of what could be said when speaking about machine learning in the context of OSA. It is important to have a holistic picture which captures co-morbidities and rules out any other conditions that are symptomatically similar to OSA. It was also discussed that there is a “need for a human operator to help”(P4) with the complementary analysis and supervision of machine learning. This protects against any errors or shortcomings from machine learning detection systems. What further supports the above, is that P5 mentioned that “Machine learning will definitely help, but that will be in the future” and “AI is new and a lot of people do not understand it”, so it is important to keep human experts in the loop and ensure that “researchers and clinicians lead and not machine learning”(P5). Clinicians have this concern because the opaque, “black box” nature of many modern AI systems fundamentally clashes with the medical mandate for evidence-based, transparent decision-making. If a machine makes an error, the clinician ultimately bears the moral and legal responsibility. Therefore, the future integration of machine learning will need to rely on the development of Explainable AI; systems must be designed to show their working and present confidence levels, allowing the human oversight to easily verify the algorithm’s reasoning.

Participants were asked about the type of results they would like to see from the machine learning algorithms. Most mentioned that “AHI is fine”(P2), and that “AHI would be enough to allow me to diagnose the patient”(P8). Two participants P5 and P9 mentioned that they would be interested in “the lowest oxygen saturation”(P9). There was P10 that mentioned, “AHI can be deceiving, hypoxic burden or oxygen desaturation index might be needed in the future depending on the research and guidelines.”(P10). This shows that the AHI which is the average number of apnoeic and hypopnoeic episodes per hour, would be more than enough for clinicians’ needs. Any desire for extra, supplementary metrics indicates a shift in how OSA severity is understood. Clinicians recognise that AHI alone can oversimplify the physiological effects of OSA, treating short and long apnoeic events equally. Moving forward, machine learning developers should not restrict their models to a single AHI output. Instead, future systems must be flexible, providing a comprehensive dashboard that includes the lowest oxygen saturation and hypoxic burden to future-proof the technology against evolving clinical guidelines.

Some participants also worried about the legal aspect and whether there are official guidelines to support the use of machine learning for the detection of OSA: “I follow the guidelines, and if the guidelines change and approve a machine learning system, then I will use it”(P3). This adds to the fact that machine learning is relatively new, and there is not a lot of guidance and rules to manage its use. So, robust regulatory frameworks and standardised clinical guidelines are required to make the picture truly clear. Technical accuracy alone is insufficient for clinical adoption. From a practical standpoint, clinicians fear medical liability when using unapproved software. For future machine learning tools to transition from research novelties to daily clinical assets, developers must prioritise rigorous, peer-reviewed clinical trials and pursue formal regulatory clearance for any project. This aligns with the work reported by Cai et al., which mentions that information on the US Food and Drug Administration (FDA) regulatory approvals and peer-reviewed publications validating machine learning tools would help with adopting a diagnostic AI assistant (9).

## 5 Discussion

For clinicians to use machine learning systems for the detection of OSA, it is important that they are kept in the process in order to ensure the best healthcare service is provided to the patient. While machine learning algorithms can have high performance at diagnosing patients and detecting OSA, the interviews show that detection of OSA and the use of machine learning extend beyond signal analysis and classification. The current diagnosis remains reliant on patient history. This aligns with the observations of Jacobs et al., who noted that clinicians rely on contextual factors to reach a definitive diagnosis (5). Consequently, machine learning cannot currently operate as an autonomous diagnostic entity, and it must be used as a complementary tool that supports medical expertise, rather than replacing it. Just as Wang et al. found that clinicians prefer to rely on their own knowledge rather than to be directed by AI (8), the participants in this study maintained that clinical oversight is non-negotiable, particularly with complex patient histories and comorbidities.

Furthermore, the idea of using a reduced number of signals for the detection of OSA highlights that it is a cost-effective triage mechanism to manage waiting lists. It is a method to reserve full polysomnography for complex cases. However, clinicians point out that there are diagnostic vulnerabilities and problems with this approach. A limited-channel system struggles with artefacts, fails to definitively confirm actual sleep time without EEG, and risks missing events like RERAs. More importantly, reduced signals cannot reliably differentiate OSA from symptomatically overlapping conditions such as pulmonary embolism, Cheyne-Stokes respiration or anxiety. This reinforces the sentiment that while machine learning can optimise resource allocation through preliminary screening, it cannot replace the “gold standard” of full polysomnography without risking patient safety.

Finally, trust and regulatory clarity remain paramount barriers to adoption. The overarching clinician desire for the diagnostic picture to be “clear and not hazy” mirrors the findings of both Gu et al. (7) and Cai et al. (9). Clinicians require transparent systems and verifiable evidence before integrating AI into their workflows, and much of the observed hesitance is heavily tied to a lack of established clinical guidelines for machine learning in sleep medicine. To enable successful adoption, institutions and governments must urgently close the gap between rapid technological advancement and existing medical frameworks by providing clear laws and guidance. This regulatory adaptation is slowly taking place; for instance, regulators, including the FDA are exploring how to adapt existing frameworks and produce guidance for integrating AI and machine learning into software as a medical device (14). Establishing these regulations will make the landscape clearer for patients, clinicians, and the researchers developing these tools, which will simultaneously improve system design and boost clinical adoption. Interestingly, the industry is already responding to this shift, as software developers increasingly treat emerging regulatory frameworks and guidance as core design specifications (15). Ultimately, ensuring that machine learning systems achieve official regulatory validation will be the vital step in transforming these algorithms from experimental models into trusted clinical decision-support systems.

## 6 Limitations and future work

This work has several limitations that must be acknowledged. First, the small sample size of ten participants means the results are only preliminary. While the study findings offer in-depth insights into a specific group, it inherently suffers from limited generalisability. The perspectives may not fully represent clinicians across different healthcare systems and medical specialities. Additionally, there is a potential for selection bias, as the clinicians who volunteered to participate may naturally have a stronger interest in, or stronger opinions about, technology and artificial intelligence compared to the broader clinical population. The study is also susceptible to interviewer bias. This is a common limitation in semi-structured qualitative research—where the interviewer’s framing of the questions or probing techniques could inadvertently influence participant responses. Finally, while this study successfully captures clinician perspectives, it does not consider the perspectives of patients or their carers. As highlighted in our findings, the observations of sleep partners are often a critical catalyst in suspected OSA diagnoses. As patients and caregivers are major stakeholders in any diagnostic process, this absence limits the comprehensive understanding of how AI integration might affect patient trust and the overall care experience.

To address the limitations, future work will focus on expanding the study by recruiting at least ten additional participants, which is expected to produce more diverse themes and validate the preliminary results. The interview questions will also be refined based on the insights gained from the initial cohort. Furthermore, future research will aim to run a co-design workshop where a prototype of the machine learning system will be introduced to participants. Gathering direct feedback on a tangible prototype will be crucial for improving the design of machine learning as a clinical decision-support system for OSA detection. Lastly, subsequent phases of this research must incorporate qualitative studies focused on patients and caregivers to ensure the technology is acceptable to all end-users in the clinical pathway.

## 7 Conclusion

This study has used interviews (n=10) to explore the perspectives of clinicians on the use of machine learning for the detection of OSA. Findings suggest that overall there are mixed opinions on the use of machine learning in the field of OSA. Some would like the use of multiple signals rather than 1 signal, for example. Clinicians also require clear guidelines on the use of machine learning in the medical setting. Also, clinical oversight is highlighted and the fact that there always needs to be a human with medical expertise being involved in the diagnosis, especially if patients have a complex medical history and co-morbidities.

## Data Availability

The datasets presented in this article are not readily available because the participants of this study did not give written consent for their data to be shared publicly, so due to the sensitive nature of the research supporting data is not available. Requests to access the datasets should be directed to ze18454@bristol.ac.uk.
